# Effect of lycopene on the copper-induced oxidation of low-density lipoprotein in broiler chickens

**DOI:** 10.1186/s40064-016-2035-6

**Published:** 2016-03-31

**Authors:** Kyung-Woo Lee, Won-Don Choo, Chang-Won Kang, Byoung-Ki An

**Affiliations:** Laboratory of Poultry Nutrition, Department of Animal Science and Technology, College of Animal Bioscience and Technology, Konkuk University, 120 Neungdong-ro, Gwangjin-gu, Seoul, 143-701 South Korea

**Keywords:** Antioxidant, Broiler chickens, Growth performance, Lycopene

## Abstract

The present study was undertaken to investigate whether dietary lycopene (LP) could prevent the copper-mediated oxidation of low density lipoprotein (LDL), and affect growth performance, relative organ weights, plasma and meat lipid profiles, and LP contents in plasma and tissues in broiler chickens. A total of 160 day-old male broiler chicks were randomly allotted into 16 pens with rice husk as a bedding material. Each experiment had 4 replicates, 10 chicks per replicate (n = 4 per treatment). A corn-soybean meal base diet was used as a control diet (CONT). To formulate the experimental diets, the base diet was added with LP at the levels of 10 (LP10) or 20 mg/kg (LP20), or 17 g/kg of tomato paste (TP17) which was equivalent to 5 ppm of LP. The experiment lasted 4 weeks. Growth performance and relative organ weights were not affected (*p* > 0.05) by any of dietary treatments. Dietary LP significantly lowered (*p* < 0.05) triglyceride and LDL cholesterol at 2 weeks of age, but did not affect them at 4 weeks of age. Total cholesterol in thigh meats was not altered by dietary treatments. LP was not detected in leg meats in all chicks, nor in liver or plasma of the CONT group. LP was found in liver and plasma, being the former greater in the concentration, of the chicks fed diets containing LP10, LP20, or TP17. At 2 and 4 weeks, the copper-mediated oxidation of LDL was delayed (*p* < 0.05) in either LP- or TP-fed chickens compared with the CONT group. In conclusion, LP lowers triglyceride and LDL cholesterol, is deposited into serum and liver, and prevents the LDL oxidation in broiler chickens, confirming the role of LP in the lipid-lowering and antioxidant properties in broiler chickens.

## Background

Lycopene (LP) is an aliphatic hydrocarbon, a bright red pigment, which is a naturally present carotenoid in fruits and vegetables. Tomatoes are known to be the major source of LP with the content of 3100–8600 µg per 100 g of tomatoes or their products (Stahl and Sies 1996). The most well-known biological effects of LP intake are to act as the antioxidant or hypocholesterolemic agent (Di Mascio et al. 1989). It has been known that the hypocholesterolemic or triglyceride-lowering effect of LP is attributed to inhibition of de novo cholesterol synthesis and lipogenesis (Chung et al. 2012; Palozza et al. 2012). Indeed, Fuhrman et al. (1997) proved that LP suppressed the cholesterol synthesis from acetate by 73 % using the macrophage cell line, and confirmed in vitro observation in healthy males in vivo that the concentration of plasma low-density lipoprotein (LDL) cholesterol was reduced by 14 % by LP intake for 3 months. In studies with chickens, dietary LP has been known to improve meat quality via inhibiting lipid peroxidation and also to lower serum lipids. For example, Sun et al. (2015) observed that dietary LP lowered malondialdehyde (MDA) content in liver with enhanced antioxidant enzyme system. In line with the well-established role as natural antioxidant, dietary LP significantly lowered MDA concentration in cooked or raw breast meats stored at refrigeration or 4 °C and retarded the iron-induced lipid oxidation in raw breast meat samples (Botsoglou et al. 2004). Of interest, when chickens were exposed to various stressors such as heat or challenge with T-2 toxin or lipopolysaccharide, dietary LP has been known to enhance hosts’ antioxidant systems and improve the oxidative stability of meats (Leal et al. 1999; Sahin et al. 2006, 2008; Sun et al. 2014a). In clinical trial with human, LP has been known to inhibit the LDL oxidation with decreasing risk of atherosclerosis and coronary heart disease in human (Agarwal and Rao 1998; Fuhrman et al. 2000; Basu and Imrhan 2007). It is reported that LP absorbed is transported mainly by LDL, which primarily accumulate in liver, seminal vesicles and prostate tissue (Palozza et al. 2012). It is however that LP effect on LDL oxidation which had been reported in human has not been tested in chickens, which prompted us to test whether dietary LP could play a role in inhibiting LDL oxidation in broiler chickens. In addition, production traits, lipid profile in blood and meat, and presence of LP in blood, liver and thigh meats were monitored. Previously, it was reported that dietary LP increased in villus height and villus height:crypt depth ratio and enhanced growth performance in broiler chickens (Sevcikova et al. 2008; Sun et al. 2015).

## Methods

### Animals, diets and experimental design

A total of 160 day-old feather-sexed male chicks (Ross) were purchased from local hatchery. Upon arrival, they were individually weighed, randomly housed into one of 16 pens with rice husks as a bedding material and subjected to one of four experimental diets. Each treatment had 4 replicate, 10 chicks per replicate (n = 4 replicates/treatment). There were four dietary treatments: corn-soybean meal base control diet (CONT); CONT + 10 mg/kg of lycopene (LP10); CONT + 20 mg/kg of lycopene (LP20); and CONT + 17 g/kg of tomato paste (TP17), respectively. Lycopene (DSM Nutritional Products Inc., Basel, Switzerland) and tomato paste (Heinz) was commercially available and the latter contained 300 mg of lycopene per kg according to the manufacturer’s specification. Corn-soybean meal base diet (Table 1) was used as a CONT diet. The lycopene-enriched diets were formulated by supplementing the CONT diet with either 10 or 20 mg/kg of synthetic lycopene or 17 g/kg of tomato paste (equivalent to provide 5 mg/kg of lycopene) at the expense of equal amount of the CONT diet. Experimental diets were prepared weekly. Feed and water were provided ad libitum. The temperature of facility was maintained at 32 °C during the first week and gradually decreased to reach 23 °C at 3 weeks and kept thereafter. All experimental protocols were approved by the Animal Care Committee of KonKuk University.

**Table 1:** Ingredients and composition of the basal diet

Ingredients (%)	0–14 days	15–28 days
Corn	55.88	65.73
Wheat	8.00	–
Soybean meal	23.00	21.50
Dehulled soybean meal	0.30	0.30
Rapeseed meal	3.00	4.00
Corn gluten meal	4.70	2.70
Yellow grease	1.20	2.40
Limestone	0.50	0.60
Tricalcium phosphate	2.10	2.00
Salt	0.22	0.19
DL-methionine	0.15	0.10
Lysine HCl	0.71	0.26
Choline-Cl	0.10	0.08
Vitamin and mineral premix^a^	0.14	0.14
Total	100.0	100.0
Calculated composition		
Crude protein (%)	20.5	18.5
Crude fat (%)	3.47	4.85
Crude fiber (%)	3.56	3.65
Crude ash (%)	5.48	5.33
Ca (%)	0.97	0.96
Available P (%)	0.45	0.43
MEn (kcal/kg)	2944	3026

^a^Vitamin and mineral premix provided following nutrients per kg of diet: vitamin A, 19,000 IU; vitamin D3, 5000 IU; vitamin E, 50 mg; vitamin K_3_, 5.25 mg; thiamin, 3.50 mg; riboflavin, 14 mg; pyridoxine, 7 mg; cobalamin, 0.027 mg; niacin, 146.0 mg; biotin, 0.21 mg; folic acid, 1.75 mg; pantothenic acid, 22.85 mg, Fe, 72 mg; Mn, 90 mg; Zn, 74 mg; I, 1.8 mg; Se, 0.36 mg; Cu, 4.8 mg

### Sampling

Feed intake and body weight per pen were measured on a weekly basis and used to calculate feed conversion ratio. At 14 and 28 days, blood was collected into Vacutainer tubes containing K_3_-EDTA (1 mg/mL) from three birds per replicate after cervical dislocation. Plasma was obtained by gentle centrifugation at 4 °C, allotted into 4 tubes, and stored at −70 °C until prior to use. Immediately after blood sampling, liver and spleen were excised, weighed and expressed as relative weight to live body weight. At 28 days, thigh meat, abdominal fat, and bursa of Fabricius also sampled. Liver and thigh meat sampled were washed with ice-cold phosphate buffered saline, blotted to dry on towel, and stored at −70 °C until use.

### Measurements of total cholesterol, triglyceride, and lipoproteins in plasma

Total cholesterol, triglyceride, and high-density (HDL) and low-density lipoprotein (LDL) in plasma were analyzed by automatic blood analyzer (Hitachi 7600-110, 7170).

### Measurement of total cholesterol in thigh meats

Total cholesterol in thigh meat samples was extracted by direct saponification procedure as described by Adams et al. (1986) and quantified by gas chromatography on a Hewlett Packard 5890 series II (Avondale, PA) equipped with flame ionization detector and a SAC™-5 capillary column (30 m × 0.25 mm i.d., 0.25 µm film thickness, Supelco, Bellefonte, PA). The carrier gas was helium and the initial and terminal column temperature was programmed at 280 °C. The detector was set at 300 °C.

### Measurement of lycopene concentration in plasma, liver and thigh meat

Total lycopene in plasma, liver and thigh meat sampled at 2 and 4 weeks were extracted by the method of Boileau et al. (2000) and analyzed by the method of Wei et al. (2001) using high performance liquid chromatography (HPLC). In brief, approximately, 0.1 g of tissue samples was minced thoroughly, dissolved in 6 mL of a potassium hydroxide/ethanol (1:5) solution containing 1 g/L butylated hydroxytoluene (BHT) and vortexed. Tissues were saponified at 60 °C for 30 min. Lycopene was extracted twice under yellow light using equal volumes of hexane (6 mL) plus 2 mL distilled water. Extracts were dried in a speedvac concentrator (Eppendorf, Hamburg, Germany) and stored at −20 °C for no longer than 2 days before HPLC analysis. For measurement of LP in plasma, to 500 µL of plasma was added with the equal amount of ethanol containing 100 g/L BHT and vortexed. Plasma was then extracted twice under yellow lights using 1.0 mL hexane. Extracts were dried and stored as described above.

The HPLC system included Waters 510 pumps, a Waters 717 plus auto sampler, a Waters 486 Tunable Absorbance detector, and Waters Nova-Pak (5 µm, 3.9 cm × 300 mm) C18 column. The mobile phase was methanol:acetonitrile:chlorform (47:47:6, v/v/v) and the flow rate was 1.0 mL/min. The HPLC was controlled by Waters Millennium chromatography software and the lycopene peak was monitored at 472 nm. Lycopene concentration was calculated using a calibration curve prepared with the pure lycopene standard (L-9879, Sigma Co., St. Louis, MO, USA).

### Isolation and copper-mediated oxidation of LDL

LDL was isolated from plasma by a single density gradient ultracentrifugation as described by Terpstra et al. (1981) and the susceptibility of LDL isolated to in vitro oxidation by copper ions was evaluated according to the procedure described by Wallin et al. (1993). In brief, freshly sampled plasma (1000 µL) was taken in a centrifuge tube (thin-walled polyallomer, 13 × 51 mm, Beckman Instruments), and the density was adjusted to 1.3 g/mL by the addition of solid 0.1140 g of potassium bromide (KBr) and 0.025 g of sucrose. Then the tube was gently over layered with 2 mL of a salt solution with a density of 1.1 g/mL [11.42 mg of sodium chloride (NaCl) and 75.98 mg of KBr/mL], which then was overlaid with 2 mL of distilled water to fill the tube. Centrifugation (Optima XL-100 K, Beckman) was performed at 280,000*g* at 4 °C for 7 h. The LDL fraction was collected using a syringe by inserting the needle directly into the LDL band and applying gentle suction. The isolated LDLs were desalted by dialysis against 10 mM sodium phosphate buffer (pH 7.4) containing 150 mM NaCl at 4 °C. The protein content of the LDL was determined by the modified Lowry method (Markwell et al. 1978) with bovine serum albumin used as a standard.

Ex vivo LDL oxidation was carried out in a 96-well microtiter plate at 37 °C. Seven replicate plates were prepared to observe the time-dependent kinetics on the LDL oxidation. LDL isolated was diluted with 10 mM Hepes buffer containing 2 mM of magnesium chloride, 4 mM of calcium chloride and 150 mM of NaCl (pH 7.2). 100 µL of diluted LDL solution (25 µg protein) was added with 10 µL of 55 µM of coper sulfate to initiate the oxidation. One plate, which had not been added with copper sulfate, but added with 10 µL of 1 mM BHT was served as the baseline value (zero time point). The copper-added plates were incubated at 37 °C in a slowly shaking bath covered with adhesive polyester film permeable to air. Incubation periods varied from 0 to 240 min. At the indicated time point, the plate was added with 10 µL of 1 mM BHT to terminate the reaction.

The oxidation of plasma LDL was assessed by measuring thiobarbituric acid-reactive substances (TBARS) using the method of Wallin et al. (1993). Briefly, the plate was added with 70 µL of Herpes buffer, 50 µL of 50 % trichloroacetic acid, and 75 µL of 1.3 % thiobarbituric acid dissolved in 0.3 % sodium hydroxide. The plate was incubated at 60 °C for 40 min and cooled in refrigerator. To reduce turbidity, the plate was added with 10 µL of 20 % sodium dodecyl sulfate and the absorbance was measured as the difference between 530 and 600 nm. The concentration of TBARS was expressed as nanomoles of malondialdehyde equivalents (MDA) per milligram LDL protein using freshly diluted 1,1,3,3-tetraethoxypropane for the standard curve.

### Statistical analysis

Data obtained in this study were evaluated by one-way analysis of variance using general linear model procedure of SAS (SAS 1986). If the F test for treatment effect was significant, differences between treatment means were determined using the Duncan’s multiple range test (Duncan 1955). The pen was considered an experimental unit and significance was determined at *p* < 0.05.

## Results

**Fig. 1:**
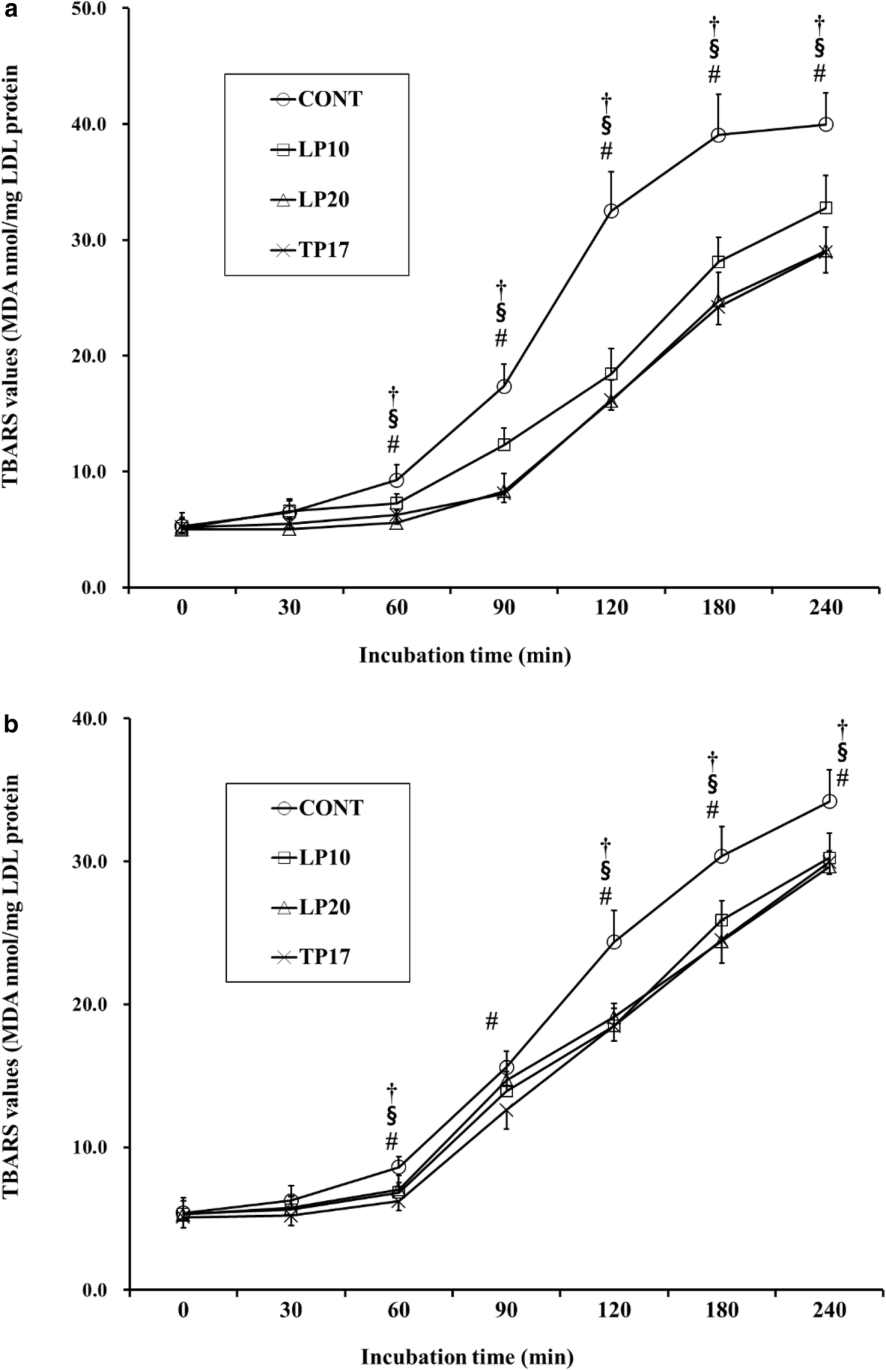
Effect of lycopene on the copper ion-induced low density lipoprotein oxidation in broiler chickens. Low-density lipoprotein (LDL) isolated from 2 (**a**) or 4 (**b**) week-old chickens were subjected to ex vivo oxidation in the presence of copper ions and the concentration of thiobarbituric acid-reactive substances (TBARS) formed were measured as the absorbance difference between 530 and 600 nm at the indicated time points, and expressed as nM of malondialdehyde equivalents (MDA) per mg LDL protein using freshly diluted 1,1,3,3-tetraethoxypropane for the standard curve. Values are mean TBARS values from 4 replicates ± SE. The *dagger* (†) denotes *p* < 0.05 LP10 versus CONT; the *section mark* (§) denotes *p* < 0.05, LP20 versus CONT; the *number sign* (#) denotes *p* < 0.05, TP17 versus CONT

Production traits (Table 2), i.e., weight gain, feed intake and feed:gain ratio and relative organ weights (Table 3) were not affected by dietary treatments. At 2 weeks of age, plasma triglyceride concentration was significantly lowered (*p* < 0.05) by chicks fed LP10 or LP20 diets compared with the control counterparts (Table 4). LDL cholesterol was significantly low (*p* < 0.05) in LP20-fed chickens compared with the CONT group. However, total and HDL cholesterols were not affected (*p* > 0.05) by dietary treatments. At 4 weeks of age, none of lipid profiles measured were affected by any of dietary LP treatments. In addition, total cholesterol content in thigh meat was not different across the treatments (Table 4). At 4 weeks, LP was not detected in leg meats in all treated groups, nor in plasma and liver of the control diet-fed chickens (Table 5). LP was significantly elevated (*p* < 0.05) in liver and plasma compared with the CONT group, having the LP20-fed chicks the highest LP content. The effect of LP supplementation on the copper-mediated LDL oxidation as measured by formation of TBARS concentration is presented in Fig. 1. At 2 weeks (panel A in Fig. 1), the extent of LDL oxidation was significantly higher (*p* < 0.05) in the control chicks compared with those fed LP- or TP-enriched diets. The LP- or TP-mediated retardation in LDL oxidation was apparent at 60 min of incubation and kept thereafter. At 4 weeks (panel B in Fig. 1), similar pattern as shown in 2 weeks was observed, being the LDL oxidation significantly lower in the LP- or TP-treated group versus the CONT group.

**Table 2:** Effect of lycopene on growth performance in broiler chickens

Items	CONT	LP10	LP20	TP17
Weight gain (g/day/bird)	25.9 ± 0.49	27.0 ± 0.72	26.7 ± 0.37	26.0 ± 0.07
Feed intake (g/day/bird)	46.4 ± 0.22	48.4 ± 0.57	48.0 ± 0.53	47.5 ± 0.94
Feed:gain ratio (g/g)	1.79 ± 0.04	1.79 ± 0.03	1.80 ± 0.02	1.82 ± 0.03

Values reported as mean ± SE (n = 4/treatment)

CONT = no-added basal diet, LP10 = lycopene at 10 ppm in basal diet; LP20 = lycopene at 20 ppm in basal diet; TP17 = tomato paste at 1.7 % in basal diet

**Table 3:** Effect of lycopene on relative organ weights in broiler chickens

Organs (g/100 g BW)	CONT	LP10	LP20	TP17
2 weeks				
Liver	2.68 ± 0.06	2.79 ± 0.05	2.67 ± 0.06	2.66 ± 0.04
Spleen	0.06 ± 0.01	0.06 ± 0.01	0.06 ± 0.01	0.06 ± 0.01
4 weeks				
Liver	1.80 ± 0.05	1.89 ± 0.04	1.82 ± 0.04	1.82 ± 0.04
Spleen	0.08 ± 0.00	0.09 ± 0.01	0.07 ± 0.01	0.08 ± 0.01
Abdominal fat	1.54 ± 0.10	1.54 ± 0.08	1.44 ± 0.08	1.44 ± 0.06
Bursa of Fabricius	0.30 ± 0.02	0.26 ± 0.03	0.28 ± 0.02	0.27 ± 0.03

Values reported as mean ± SE (n = 4/treatment)

CONT = no-added basal diet, LP10 = lycopene at 10 ppm in basal diet; LP20 = lycopene at 20 ppm in basal diet; TP17 = tomato paste at 1.7 % in basal diet

*BW* body weight

**Table 4:** Effects of lycopene on plasma lipids and cholesterol contents in thigh meats of broiler chickens

	CONT	LP10	LP20	TP17
2 weeks				
Triglycerides (mg/dL)	33.7 ± 1.67^a^	26.0 ± 1.15^b^	25.2 ± 2.04^b^	30.7 ± 2.16^a,b^
Total cholesterol (mg/dL)	156.7 ± 7.04	145.8 ± 6.37	138.2 ± 6.18	154.7 ± 7.92
HDL cholesterol (mg/dL)	116.0 ± 5.37	112.2 ± 5.09	105.9 ± 3.89	119.1 ± 6.09
LDL cholesterol (mg/dL)	34.0 ± 1.57^a^	30.3 ± 1.56^a,b^	27.3 ± 2.49^b^	29.0 ± 1.93^a,b^
4 weeks				
Triglycerides (mg/dL)	27.0 ± 1.03	32.5 ± 2.92	24.5 ± 1.55	27.0 ± 1.65
Total cholesterol (mg/dL)	157.7 ± 1.20	160.2 ± 3.42	151.8 ± 6.25	166.6 ± 2.62
HDL cholesterol (mg/dL)	107.4 ± 2.64	110.9 ± 2.76	102.2 ± 7.29	114.0 ± 4.38
LDL cholesterol (mg/dL)	51.0 ± 3.70	45.5 ± 1.18	56.5 ± 3.54	58.3 ± 6.63
Total cholesterol (mg/g of leg meat)	1.01 ± 0.04	0.93 ± 0.06	0.94 ± 0.05	0.90 ± 0.02

Values reported as mean ± SE (n = 4/treatment)

CONT = no-added basal diet, LP10 = lycopene at 10 ppm in basal diet; LP20 = lycopene at 20 ppm in basal diet; TP17 = tomato paste at 1.7 % in basal diet

*HDL* high-density lipoprotein, *LDL* low-density lipoprotein

^a,b^Values in the same row not sharing a common superscript differ significantly (*p* < 0.05)

**Table 5:** Lycopene contents in plasma and tissues of broiler chickens fed diets containing lycopene for 4 weeks

	CONT	LP10	LP20	TP17
Leg meat (µg/g)	ND	ND	ND	ND
Liver (µg/g)	ND	1.667 ± 0.347^b^	3.687 ± 0.808^a^	0.197 ± 0.054^c^
Plasma (µg/mL)	ND	0.103 ± 0.020^a^	0.122 ± 0.015^a^	0.038 ± 0.007^b^

Values reported as mean ± SE (n = 4/treatment)

CONT = no-added basal diet, LP10 = lycopene at 10 ppm in basal diet; LP20 = lycopene at 20 ppm in basal diet; TP17 = tomato paste at 1.7 % in basal diet

*ND* not detected

^a–c^Values in the same row not sharing a common superscript differ significantly (*p* < 0.05)

## Discussion

LP is a carotenoid that represents the red color of many fruits and vegetables, being rich in tomato and its products. Thus, LP has been used as natural food coloring agents and also known to benefit hosts health by consumption due to its strong antioxidants. The latter biological property of LP led to many previous studies to test whether the incorporation of LP or tomato-related byproducts as the source of LP into the diet of chickens could affect growth performance, lipid metabolism, and oxidative stability of meats. In this study, we further explored whether LP could affect the copper-mediated LDL oxidation in broiler chickens.

It is expected that dietary LP could improve growth performance in broiler chickens based on previous report on positive effect on gut physiology, i.e., increase in villus height and villus height:crypt depth ratio in broiler chicks (Sun et al. 2015). In contrast to our expectation, either of dietary LP or TP did not affect body weight gain, feed intake and feed:gain ratio in broiler chicks. It has been shown that positive or no effect of dietary LP on growth performance has been reported with chickens. For example, Sevcikova et al. (2008) reported that dietary LP at 100 mg/kg of diet, but not at 50 mg/kg of diet, significantly increased the body weight in broiler chickens compared with those fed the LP-free diet. On the other hand, dietary LP failed to affect growth performance of broiler chickens fed diet containing tomato skin extracts, which contained 200 mg of LP per kg of diet (Marzoni et al. 2014), or LP at 500 ppm (Pozzo et al. 2013). Leal et al. (1999) reported that LP feeding reversed the T-2 toxin-induced depression in feed intake and weight gain in chicken. Similarly, dietary LP did not affect growth performance in thermo-neutrally raised chickens, but significantly increased it in those exposed to heat stress (Sahin et al. 2006, 2008). Thus, it is likely that LP-mediated increase in growth performance is frequently observed in chicks raised under stressed conditions, or higher LP doses would be needed to observe, if any, the effect in non-stressed rearing condition, which was not the case in this study.

In this study, triglyceride and LDL cholesterol measured at 14 days of age was significantly lowered in chicks fed on LP20 compared with the CONT group, but the LP-mediated effect was not observed at 28 days of age. It appears that the sensitivity of plasma lipids to dietary LP changes with the age of the birds. Unfortunately, whether the difference in plasma lipids at different time points is indeed age-dependent is not clear at this stage. It has been known that the hypocholesterolemic or triglyceride-lowering effect of LP is attributed to inhibition of de novo cholesterol synthesis and lipogenesis (Chung et al. 2012; Palozza et al. 2012). However, inconsistent effect of LP on lipid metabolism has been reported for poultry. For example, it was shown that graded levels of dietary LP did not affect triglyceride and LDL cholesterol during the 35-day experimental period, but significantly increased HDL cholesterol in chicks at 35 day (Sun et al. 2014a). Sevcikova et al. (2008) failed to observe the hypocholesterolemic effect of LP in broiler chickens when LP was added into the diet at the level of 50 or 100 mg/kg of diet. According to the study by Sun et al. (2015), dietary LP increased HDL cholesterol, but did not affect total or LDL cholesterol in chickens. The latter study observed that LP feeding decreased triglyceride by 11.6 % compared with the CONT group, but the difference was not statistically different. In another trial by Sun et al. (2014b), they reported that dietary LP at 20 mg/kg of diet lowered serum total cholesterol by 23.9 %, but increased HDL cholesterol by 41.6 % in breeding hens compared with those fed the LP-free diet. Thus, the explanation on the disparate results on blood lipid profiles by LP in chickens is not readily available, but it is not likely to be caused by added doses, types of breeds used, or duration of feeding. With regard to the cholesterol content in poultry meat, it is known that the demand on cholesterol in meats is primarily met by synthesis in muscle cells (Dinh et al. 2011). In this study, total cholesterol in thigh meat measured at 28 days was not affected by either of dietary treatments, which is in line with earlier study by Rozbicka-Wieczorek et al. (2014) who reported that dietary LP at 12 ppm did not lower the cholesterol content in thigh meat. On the other hand, a significant decrease in cholesterol content in leg meat of chicks fed diet at the level of 75 ppm was observed (Englmaierova et al. 2011). Thus, the possibility of higher dietary LP levels on lowering the cholesterol content in meats cannot be excluded.

It is reported that LP is transported mainly by LDL and the absorbed LPs are known to primarily accumulate in liver, seminal vesicles and prostate tissue in rodents (Palozza et al. 2012). The latter finding explains the failure to find the presence of LP in leg meats compared with that in liver and plasma as shown in this study. Nonetheless, Pozzo et al. (2013) reported that trace amounts of LP were detected in breast (0.10 mg/kg of fresh weight) and thigh meats (0.42 mg/kg of fresh weight) of broiler chickens fed LP-added diet at the level of 500 mg. Due to the lack of studies measuring LP in poultry meats, further studies with graded levels of LP are warranted to prove the observed differences. In contrast, the presence of LP in serum and liver has been well established in poultry (Sahin et al. 2006; Englmaierova et al. 2011; Sun et al. 2014a, 2015), which corroborates well with our current study.

As LP can incorporate into LDL particle, it can play a role in preventing the oxidation of LDL (Agarwal and Rao 1998; Fuhrman et al. 2000; Muller et al. 2015). On the contrary, Carroll et al. (2000) reported that LP contents in LDL and HDL, being the former greater than the latter, were significantly increased, but the copper-mediated LDL oxidation was not affected. In this study, we observed that broiler chicks fed either LP or TP exhibited significantly low LDL oxidation at 14 and 28 days compared with those fed the CONT diet. Dietary TP (equivalent 5 ppm of LP) similarly retarded the LDL oxidation to the extents revealed by LP10 or LP20. Tomato contains various carotenoids, rich in LP, but also contains vitamin C, vitamin E, β-carotene, lutein, or zeaxanthin (Gama et al. 2006). Thus, the observed effect of TP on the oxidation can be explained by additional natural antioxidants present in tomato paste. Indeed, Fuhrman et al. (2000) reported that LP in combination with different antioxidants (i.e., vitamin E, flavonoids, garlic, β-carotene) synergistically inhibited LDL oxidation in vitro and confirmed the synergism in vivo with human subjects.

## Conclusions

In conclusion, dietary LP did not affect growth performance and relative organ weights, but lowered plasma triglyceride and LDL concentration in broiler chickens. LP was detected in serum and liver of chickens fed diets containing LP 10, LP20 or TP17, but not in the control-diet fed chickens. LP was not detected in the leg meats of all treated groups. Finally, the copper-mediated LDL oxidation was significantly retarded in broiler chickens fed LP10, LP20 or TP17 compared with those fed the control-diet. Collectively, our study provides the functional role of LP in broiler chickens. In our knowledge, this is the first report to show that addition of LP sourced either from synthetic form or tomato product into the diets of broiler chickens could delay the copper-associated LDL oxidation, probably via incorporation of LP into LDL lipoprotein.
